# Supplementary material for: Do people with symptoms of an infectious illness follow advice to stay at home? Evidence from a series of cross-sectional surveys about presenteeism in the UK
Source: BMJ Open. 2022 May 30;12(5):e060511. doi: 10.1136/bmjopen-2021-060511 (PMC9152621; doi:10.1136/bmjopen-2021-060511)
Supplement: Supplementary data [file bmjopen-2021-060511supp001.pdf]

SUPPLEMENTARY MATERIALS

Table 1. Socio-demographic characteristics associated with leaving home for different reasons while symptomatic.

Attribute	Level	*Been out to…* the shops for groceries/pharmacy [a] aOR for going out (95% CI) †	*p*	the shops for things other than groceries/pharmacy [b] aOR for going out (95% CI) †	*p*	go to work [c] aOR for going out (95% CI) †	*p*	meet friends or family that you don't live with [d] aOR for going out (95% CI) †	*p*	provide help or care for a vulnerable person [e] aOR for going out (95% CI) †	*p*
Survey wave in timepoint	20 to 22 September 2021 (wave 58)	Ref	-	Ref	-	Ref	-	Ref	-	Ref	-
	4 to 6 October 2021 (wave 59)	0.47 (0.26 to 0.84)	0.01	0.39 (0.22 to 0.70)	0.001*	0.44 (0.21 to 0.90)	0.02	0.48 (0.27 to 0.86)	0.01	0.43 (0.24 to 0.79)	0.007
	18 to 20 October 2021 (wave 60)	0.37 (0.21 to 0.68)	0.001*	0.41 (0.23 to 0.75)	0.003	0.32 (0.16 to 0.65)	0.002*	0.37 (0.21 to 0.67)	0.001*	0.35 (0.18 to 0.65)	0.001*
	1 to 4 November 2021 (wave 61)	0.55 (0.30 to 1.00)	0.05	0.52 (0.29 to 0.93)	0.03	0.33 (0.16 to 0.69)	0.003	0.41 (0.23 to 0.73)	0.002*	0.32 (0.17 to 0.61)	0.001*
	Overall	$\chi^2(3)$=11.3	0.01	$\chi^2(3)$=12.4	0.006	$\chi^2(3)$=12.3	0.006	$\chi^2(3)$=13.4	0.004	$\chi^2(3)$=16.0	0.001*
Region	North (North West, North East, Yorkshire and the Humber)	Ref	-	Ref	-	Ref	-	Ref	-	Ref	-
	Midlands (East Midlands, West Midlands, East of England)	0.89 (0.50 to 1.58)	0.69	0.97 (0.54 to 1.75)	0.93	0.93 (0.45 to 1.92)	0.84	0.75 (0.42 to 1.34)	0.34	0.59 (0.31 to 1.13)	0.11
	London	1.33 (0.68 to 2.61)	0.40	1.51 (0.77 to 2.95)	0.23	1.71 (0.74 to 3.97)	0.21	1.19 (0.62 to 2.29)	0.61	0.75 (0.37 to 1.53)	0.43
	South (South East, South West)	1.54 (0.81 to 2.92)	0.18	1.21 (0.64 to 2.28)	0.55	1.79 (0.82 to 3.91)	0.14	0.92 (0.49 to 1.72)	0.80	1.02 (0.51 to 2.01)	0.96
	Scotland, Wales, Northern Ireland	0.78 (0.41 to 1.49)	0.46	0.67 (0.34 to 1.30)	0.23	0.74 (0.32 to 1.70)	0.47	0.62 (0.32 to 1.20)	0.16	0.38 (0.17 to 0.83)	0.01
	Overall	$\chi^2(4)$=5.4	0.25	$\chi^2(4)$=5.2	0.26	$\chi^2(4)$=6.7	0.15	$\chi^2(4)$=4.3	0.37	$\chi^2(4)$=8.4	0.08
Gender	Male	Ref	-	Ref	-	Ref	-	Ref	-	Ref	-
	Female	0.78 (0.52 to 1.17)	0.22	0.75 (0.49 to 1.13)	0.16	0.55 (0.33 to 0.93)	0.03	1.09 (0.73 to 1.65)	0.67	0.67 (0.43 to 1.07)	0.09
Age (per decade)	Raw age	1.00 (0.98 to 1.02)	0.65	0.99 (0.97 to 1.01)	0.30	0.96 (0.94 to 0.99)	0.01	0.98 (0.96 to 1.00)	0.04	0.97 (0.95 to 0.99)	0.01
Age: quadratic (age-mean)^2	-	1.0001 (0.9996 to 1.0007)	0.65	1.0005 (0.9998 to 1.0012)	0.14	1.0011 (0.9998 to 1.0024)	0.11	1.0005 (0.9999 to 1.0011)	0.09	1.0004 (0.9998 to 1.001)	0.16
Dependent child in household	None	Ref	-	Ref	-	Ref	-	Ref	-	Ref	-
	Child present	1.20 (0.76 to 1.90)	0.43	1.78 (1.11 to 2.84)	0.02	2.50 (1.38 to 4.52)	0.003	1.64 (1.03 to 2.59)	0.04	2.85 (1.70 to 4.78)	<0.001*
Employment status	Not employed	Ref	-	Ref	-	-	-	Ref	-	Ref	-
	Employed, can work from home all the time	1.39 (0.81 to 2.39)	0.23	1.51 (0.87 to 2.63)	0.14	Ref	-	1.41 (0.83 to 2.42)	0.21	1.70 (0.93 to 3.12)	0.08
	Employed, cannot work from home all the time	1.00 (0.60 to 1.65)	0.99	1.24 (0.73 to 2.09)	0.43	1.26 (0.75 to 2.09)	0.38	0.80 (0.48 to 1.34)	0.40	1.39 (0.77 to 2.51)	0.27
	Overall	$\chi^2(2)$=2.2	0.33	$\chi^2(2)$=2.2	0.34	-	-	$\chi^2(2)$=5.5	0.06	$\chi^2(2)$=3.0	0.22
Socio-economic grade‡	ABC1	Ref	-	Ref	-	Ref	-	Ref	-	Ref	-
	C2DE	1.06 (0.71 to 1.59)	0.77	1.08 (0.72 to 1.63)	0.71	1.84 (1.10 to 3.07)	0.02	1.44 (0.96 to 2.17)	0.08	1.27 (0.81 to 1.99)	0.31
Financial hardship	Range 3 (least) to 15 (most)	0.98 (0.91 to 1.05)	0.50	0.91 (0.85 to 0.98)	0.01	0.92 (0.85 to 1.00)	0.05	0.97 (0.91 to 1.05)	0.48	0.86 (0.79 to 0.94)	<0.001*
Ethnicity	White British	Ref	-	Ref	-	Ref	-	Ref	-	Ref	-
	White other	0.99 (0.42 to 2.35)	0.98	1.30 (0.54 to 3.11)	0.55	0.81 (0.30 to 2.22)	0.69	0.76 (0.32 to 1.80)	0.53	1.57 (0.63 to 3.91)	0.33
	Black and minority ethnicity	1.03 (0.57 to 1.85)	0.92	1.04 (0.58 to 1.86)	0.90	0.65 (0.33 to 1.31)	0.23	1.04 (0.58 to 1.84)	0.90	1.48 (0.81 to 2.72)	0.21
	Overall	$\chi^2(2)$=0.0	0.99	$\chi^2(2)$=0.4	0.84	$\chi^2(2)$=1.4	0.49	$\chi^2(2)$=0.5	0.78	$\chi^2(2)$=1.9	0.38
First language	Not English	Ref	-	Ref	-	Ref	-	Ref	-	Ref	-
	English	1.20 (0.65 to 2.19)	0.56	1.37 (0.75 to 2.52)	0.31	1.15 (0.55 to 2.40)	0.70	1.13 (0.62 to 2.07)	0.69	0.95 (0.50 to 1.79)	0.87

Living alone	Not living alone	Ref	-	Ref	-	Ref	-	Ref	-	Ref	-
	Living alone	0.80 (0.43 to 1.49)	0.49	0.75 (0.39 to 1.44)	0.39	0.70 (0.31 to 1.59)	0.40	1.06 (0.57 to 1.99)	0.85	0.67 (0.30 to 1.49)	0.32
Vaccination status	Not vaccinated	Ref	-	Ref	-	Ref	-	Ref	-	Ref	-
	1 dose	1.29 (0.65 to 2.54)	0.47	1.06 (0.55 to 2.06)	0.86	1.87 (0.81 to 4.32)	0.14	1.35 (0.70 to 2.61)	0.37	1.37 (0.69 to 2.70)	0.37
	2 doses or more	0.90 (0.54 to 1.49)	0.68	0.77 (0.46 to 1.28)	0.31	1.17 (0.63 to 2.17)	0.63	0.75 (0.46 to 1.24)	0.26	0.78 (0.46 to 1.34)	0.37
	Overall	$\chi^2(2)=1.2$	0.54	$\chi^2(2)=1.5$	0.47	$\chi^2(2)=2.2$	0.34	$\chi^2(2)=3.7$	0.16	$\chi^2(2)=2.9$	0.23
Ever had COVID-19	Think not	Ref	-	Ref	-	Ref	-	Ref	-	Ref	-
	Think yes	1.33 (0.88 to 2.01)	0.17	1.68 (1.11 to 2.55)	0.01	1.50 (0.91 to 2.49)	0.11	1.19 (0.79 to 1.79)	0.41	1.19 (0.76 to 1.86)	0.46

A) n=462 (92.8% valid responses). B) n=462 (92.8% valid responses). C) n=337 (94.7% valid responses). D) n=462 (92.8% valid responses). E) n=462 (92.8% valid responses).

† Adjusting for all other socio-demographic characteristics.

* *p*≤.002
