# Supplementary material for: Do people with symptoms of an infectious illness follow advice to stay at home? Evidence from a series of cross-sectional surveys about presenteeism in the UK
Source: BMJ Open. 2022 May 30;12(5):e060511. doi: 10.1136/bmjopen-2021-060511 (PMC9152621; doi:10.1136/bmjopen-2021-060511)
Supplement: Author's manuscript [file bmjopen-2021-060511.draft_revisions.pdf]

BMJ Open is committed to open peer review. As part of this commitment we make the peer review history of every article we publish publicly available.

When an article is published we post the peer reviewers' comments and the authors' responses online. We also post the versions of the paper that were used during peer review. These are the versions that the peer review comments apply to.

The versions of the paper that follow are the versions that were submitted during the peer review process. They are not the versions of record or the final published versions. They should not be cited or distributed as the published version of this manuscript.

BMJ Open is an open access journal and the full, final, typeset and author-corrected version of record of the manuscript is available on our site with no access controls, subscription charges or pay-per-view fees (http://bmjopen.bmj.com).

If you have any questions on BMJ Open's open peer review process please email info.bmjopen@bmj.com

BMJ Open

Do people with symptoms of an infectious illness follow advice to stay at home? Evidence from a series of cross-sectional surveys in the UK

Journal:	*BMJ Open*
Manuscript ID	bmjopen-2021-060511
Article Type:	Original research
Date Submitted by the Author:	23-Dec-2021
Complete List of Authors:	Rubin, GJ; King's College London<br>Amlot, Richard; UK Health Security Agency<br>Fear, Nicola; King's College London, ADMMH<br>Potts, Henry; University College London, Institute of Health Informatics<br>Michie, Susan; University College London, Centre for Behaviour Change<br>Smith, Louise; King's College London, Psychological Medicine
Keywords:	COVID-19, Infection control < INFECTIOUS DISEASES, Public health < INFECTIOUS DISEASES

SCHOLARONE™
Manuscripts

I, the Submitting Author has the right to grant and does grant on behalf of all authors of the Work (as defined in the below author licence), an exclusive licence and/or a non-exclusive licence for contributions from authors who are: i) UK Crown employees; ii) where BMJ has agreed a CC-BY licence shall apply, and/or iii) in accordance with the terms applicable for US Federal Government officers or employees acting as part of their official duties; on a worldwide, perpetual, irrevocable, royalty-free basis to BMJ Publishing Group Ltd ("BMJ") its licensees and where the relevant Journal is co-owned by BMJ to the co-owners of the Journal, to publish the Work in this journal and any other BMJ products and to exploit all rights, as set out in our licence.

The Submitting Author accepts and understands that any supply made under these terms is made by BMJ to the Submitting Author unless you are acting as an employee on behalf of your employer or a postgraduate student of an affiliated institution which is paying any applicable article publishing charge ("APC") for Open Access articles. Where the Submitting Author wishes to make the Work available on an Open Access basis (and intends to pay the relevant APC), the terms of reuse of such Open Access shall be governed by a Creative Commons licence – details of these licences and which Creative Commons licence will apply to this Work are set out in our licence referred to above.

Other than as permitted in any relevant BMJ Author's Self Archiving Policies, I confirm this Work has not been accepted for publication elsewhere, is not being considered for publication elsewhere and does not duplicate material already published. I confirm all authors consent to publication of this Work and authorise the granting of this licence.

For peer review only

Do people with symptoms of an infectious illness follow advice to stay at home? Evidence from a series of cross-sectional surveys in the UK.

Rubin GJ[1] (0000-0002-4440-0570), Amlôt R[2] (0000-0003-3481-6588), Fear NT[1] (0000-0002-5792-2925), Potts HWW[3] (0000-0002-6200-8804), Michie S[4] (0000-0003-0063-6378), Smith LE[1](0000-0002-1277-2564).

1. Department of Psychological Medicine, Institute of Psychiatry, Psychology and Neuroscience, Weston Education Centre, King's College London, London SE5 9RJ UK

2. United Kingdom Health Security Agency, Behavioural Science Team, Porton Down, Salisbury UK

3. Institute of Health Informatics, University College London, London UK

4. Centre for Behaviour Change, University College London, London UK

Correspondence to: Professor James Rubin, Department of Psychological Medicine, Weston Education Centre, Cutcombe Road, London SE5 9RJ. Email: Gideon.rubin@kcl.ac.uk.

Word count: 1553

For peer review only

For peer review only - http://bmjopen.bmj.com/site/about/guidelines.xhtml

Abstract

Objectives: To assess the percentage of people with cough, fever, or loss of taste or smell who had been to work, to shops, socialised or provided care to a vulnerable person in the ten days after developing symptoms. To investigate whether these rates differed according to the type of symptom, what the participant thought the cause of their symptoms was, and whether they had taken a COVID-19 test.

Design: Four online cross-sectional surveys.

Setting: We analysed data collected 20 September to 3 November 2021, commissioned via a market research company.

Participants: Participants were aged over 16 years and lived in the UK.

Primary outcome measures: Out-of-home activity.

Results: 498 participants reported one or more symptoms and had not had a positive COVID-19 test. Most employed participants had attended work (51.2% to 56.3% depending on the symptom, 95% CIs 42.2% to 65.6%). Rates of other behaviours ranged from 31.4% (caring for a vulnerable person after developing a cough: 95% CI 24.3% to 38.4%) to 61.5% (shopping for groceries or pharmacy after developing a cough: 95% CI 54.1% to 68.9%). There were no differences according to symptom experienced or what the participant felt might be the cause. People who had taken a COVID-19 test engaged in fewer behaviours than people who had not taken a test.

Conclusion: Most people in the UK with symptoms of an infectious disease are not following advice to stay at home, even if they believe they have an infectious illness. Reducing these rates may require a shift in our national attitude to infectious illnesses.

Keywords: behaviour, symptoms, influenza-like-illness, COVID-19

For peer review only

For peer review only - http://bmjopen.bmj.com/site/about/guidelines.xhtml

Article summary

Strengths and limitations of this study

- This study reports rates of out-of-home activity in people with symptoms of infectious illness (new onset cough, fever or loss of their sense of smell or taste).
- Despite advice from the National Health Service to stay at home if symptomatic, most people with symptoms report leaving home. Reducing these rates may require a shift in our national attitude to infectious illnesses.
- Participants reported on symptoms they had developed in the last 10 days. If symptoms developed 10 days ago, they may no longer be infectious. However, official advice for common colds and influenza indicate that people are infectious for 14 days and 8 days respectively.
- Data are self-report. Therefore, rates may be underestimates.

For peer review only

For peer review only - http://bmjopen.bmj.com/site/about/guidelines.xhtml

INTRODUCTION

The spread of infectious disease within a workplace represents a risk to employees, those who come into contact with them and to the productivity of the organisation. Although transmission can be reduced if employees who are ill stay at home, systematic reviews have demonstrated that there are a wide range of factors that determine whether someone will have the capability, opportunity or motivation to do so.[1,2]

The COVID-19 pandemic has made this issue particularly pressing. In the UK, people who develop a new continuous cough, a fever, or a loss or change to their sense of taste or smell (the so-called 'cardinal symptoms' of COVID-19) are urged to stay at home and to take a polymerase chain reaction (PCR) test. If they receive a positive test result, they are then legally obliged to remain at home. Yet self-isolating if you receive a positive COVID-19 result is only one aspect of the UK Government's plan to ease pressure on the National Health Service during the pandemic. According to official guidance, people should also "[try] to stay at home if you are feeling unwell" even in the absence of a positive COVID-19 test, while businesses are "encouraged to ask employees to stay at home if they are feeling unwell."[3] This is intended to reduce the spread of influenza-like illness throughout workplaces and reinforces pre-exiting advice to stay off work or school when ill.[4]

While a growing body of research has explored whether people are adhering to policies around COVID-19 testing and self-isolation,[5] we are unaware of any evidence as to whether people are adhering to advice to "stay at home if you are feeling unwell" even in the absence of COVID-19. In this study, we explored the behaviour of people who reported having symptoms using a series of national surveys. We analysed rates of five behaviours among people who were symptomatic: going to work, going to the shops for food or medicine, going to the shops for other things, meeting up with friends or family you do not live with, and providing help or care for a vulnerable person. We tested whether rates differed according to the symptom that was reported, what the participant believed might have caused their symptoms, and whether the participant had taken a test for COVID-19.

METHODS

Design

The English Department of Health and Social Care commissioned a series of cross-sectional, nationally representative surveys from market research companies, beginning in January 2020. For this analysis, we used data from survey waves 58 to 61 (20 September to 3 November 2021). More details are available elsewhere.[6]

Participants

Participants were aged over 16 years, lived in the UK and had previously opted-in to receive invitations to take part in online market research surveys. Quotas were used based on age and gender (combined) and region to ensure the sample was broadly representative of the UK population on these variables. Quotas were based on mid-year (2018) projections from the Office for National Statistics.

Questionnaire items

Participants were asked whether they had developed any from a list of 13 symptoms in the past ten days. This included new continuous cough, high temperature / fever, loss of sense of smell and loss of taste. Participants who reported one of these symptoms were asked "what do you think your

For peer review only - http://bmjopen.bmj.com/site/about/guidelines.xhtml

symptoms could have been caused by" and were able to tick one or more of: "hayfever / allergies," "a cold," "asthma," "flu," "coronavirus," "long COVID," "other, please specify" [with a free-text space provided] and "don't know." They were also asked which, if any, of a list of actions they took "while you had these symptoms," which included "I took a test to confirm whether I have coronavirus." Anyone who ticked this option was asked whether they took a PCR test or a lateral flow test (LFT). Participants reporting cardinal symptoms were also asked "how many times after developing symptoms recently, if at all, have you completed each of these activities" and were presented with a list of eleven daily activities, of which five might involve contact with other people.

Patient and public involvement

Lay members served on the advisory group for the project that developed our prototype survey material; this included three rounds of qualitative testing.[7] Due to the rapid nature of this research, the public was not involved in the further development of the materials during the COVID-19 pandemic.

Analysis

We restricted our sample to participants who reported having experienced one or more cardinal symptoms. We excluded anyone who reported that their most recent test for COVID-19 was positive, on the basis that such people would be legally obliged to self-isolate and should have received support and regular encouragement to do so via the UK's NHS Test and Trace service. We calculated the percentage of participants who reported engaging in each of the five activities. To calculate a percentage for work attendance, we restricted the sample to those who reported being in full, part-time or self-employment. We used χ^2 tests to assess whether attribution of symptoms was associated with behaviour, by grouping participants into mutually exclusive groups according to whether they reported COVID-19 as a possible cause, whether they reported another infectious disease as a possible cause (excluding COVID-19), or whether they did not list any infectious disease as a cause. We also assessed whether behaviour differed according to whether participants had sought a test for COVID-19 because of their recent symptoms.

RESULTS

Out of a total sample of 8547, 548 participants (6.4%) reported cardinal symptoms. Of those, 50 reported that their most recent COVID-19 test was positive and were excluded. Of the remaining 498: 54.8% (n=273) were male, 44.8% (n=223) were female and 0.4% (n=2) preferred to self-describe or not say; 71.3% (n=355) identified as white British, 7.4% (n=37) identified as white other, 20.1% (n=100) identified as being from another ethnic group and 1.2% (n=6) preferred not to say; and 71.5% (n=365) were working, 26.7% (n=133) were not working and 1.8% (n=9) preferred not to say. The mean age was 36.3 years (standard deviation 15.2 years).

Most people with fever (52.5%, 95% CI 43.5% to 61.4%), cough (51.2%, 95% CI 42.2% to 60.3%) and loss of taste or smell (56.3%, 95% CI 46.9% to 65.6%) reported having been to work after developing their symptoms. Rates of going to the shops, meeting friends or family that you do not live with, and providing care for a vulnerable person ranged from 31.4% (95% CI 24.3% to 38.4%) to 61.5% (95% CI 54.1% to 68.9%; Table 1). Rates did not differ according to what symptom was reported or what the participant believed had caused their symptoms. Participants who had taken a test to check whether they had COVID-19 were generally less likely to engage in each of the behaviours.

For peer review only - http://bmjopen.bmj.com/site/about/guidelines.xhtml

Table 1. Behaviours among people with fever, cough or loss of taste or smell and who have not had a positive COVID-19 test

Been out to…

		the shops for groceries/pharmacy					the shops for things other than groceries/pharmacy				
		No		Yes			No		Yes		
		% (95%CI)	*n*	% (95%CI)	*n*	*p*	% (95%CI)	*n*	% (95%CI)	*n*	*p*
Symptom	Fever	40.7 (33.2, 48.2)	68	59.3 (51.8, 66.8)	99	.91	48.5 (40.8, 56.2)	81	51.5 (43.8, 59.2)	86	.11
	Cough	38.5 (31.1, 45.9)	65	61.5 (54.1, 68.9)	104		55.6 (48.1, 63.2)	94	44.4 (36.8, 51.9)	75	
	Loss of taste/smell	39.1 (31.5, 46.8)	63	60.9 (53.2, 68.5)	98		44.1 (36.3, 51.9)	71	55.9 (48.1, 63.7)	90	
Attributed to[a]	COVID-19	41.6 (31.8, 51.4)	42	58.4 (48.6, 68.2)	59	.43	48.5 (38.6, 58.4)	49	51.5 (41.6, 61.4)	52	.71
	Infectious illness	40.5 (35.0, 46.0)	125	59.5 (54.0, 65.0)	184		50.8 (45.2, 56.4)	157	49.2 (43.6, 54.8)	152	
	Non-infectious illness	33.3 (23.2, 43.4)	29	66.7 (56.6, 76.8)	58		46.0 (35.3, 56.7)	40	54.0 (43.3, 64.7)	47	
Testing uptake[a]	No test	33.8 (28.4, 39.2)	100	66.2 (60.8, 71.6)	196	.001	40.9 (35.2, 46.5)	121	59.1 (53.5, 64.8)	175	<.001
	LFT only	40.2 (30, 50.4)	37	59.8 (49.6, 70)	55		60.9 (50.7, 71)	56	39.1 (29, 49.3)	36	
	PCR test	54.1 (44.6, 63.6)	59	45.9 (36.4, 55.4)	50		63.3 (54.1, 72.5)	69	36.7 (27.5, 45.9)	40	

		go to work [total n=356]					meet friends or family that you don't live with					provide help or care for a vulnerable person				
		No		Yes			No		Yes			No		Yes		
		% (95%CI)	*n*	% (95%CI)	*n*	*p*	% (95%CI)	*n*	% (95%CI)	*n*	*p*	% (95%CI)	*n*	% (95%CI)	*n*	*p*
Symptom	Fever	47.5 (38.6, 56.5)	58	52.5 (43.5, 61.4)	64	.73	50.9 (43.2, 58.6)	85	49.1 (41.4, 56.8)	82	.75	59.9 (52.4, 67.4)	100	40.1 (32.6, 47.6)	67	.17
	Cough	48.8 (39.7, 57.8)	59	51.2 (42.2, 60.3)	62		52.7 (45.1, 60.3)	89	47.3 (39.7, 54.9)	80		68.6 (61.6, 75.7)	116	31.4 (24.3, 38.4)	53	
	Loss of taste/smell	43.8 (34.4, 53.1)	49	56.3 (46.9, 65.6)	63		48.4 (40.6, 56.2)	78	51.6 (43.8, 59.4)	83		60.2 (52.6, 67.9)	97	39.8 (32.1, 47.4)	64	
Attributed to[a]	COVID-19	51.2 (40.2, 62.3)	42	48.8 (37.7, 59.8)	40	.29	53.5 (43.6, 63.4)	54	46.5 (36.6, 56.4)	47	.10	59.4 (49.7, 69.1)	60	40.6 (30.9, 50.3)	41	.70
	Infectious illness	43.9 (37.1, 50.6)	93	56.1 (49.4, 62.9)	119		52.8 (47.2, 58.3)	163	47.2 (41.7, 52.8)	146		64.1 (58.7, 69.5)	198	35.9 (30.5, 41.3)	111	
	Non-infectious illness	50.8 (37.9, 63.7)	31	49.2 (36.3, 62.1)	30		40.2 (29.7, 50.7)	35	59.8 (49.3, 70.3)	52		63.2 (52.9, 73.6)	55	36.8 (26.4, 47.1)	32	
Testing uptake[a]	No test	41.2 (34.6, 47.7)	91	58.8 (52.3, 65.4)	130	.03	44.3 (38.6, 49.9)	131	55.7 (50.1, 61.4)	165	.002	54.4 (48.7, 60.1)	161	45.6 (39.9, 51.3)	135	<.001
	LFT only	55.2 (42.0, 68.4)	32	44.8 (31.6, 58)	26		58.7 (48.4, 68.9)	54	41.3 (31.1, 51.6)	38		81.5 (73.4, 89.6)	75	18.5 (10.4, 26.6)	17	
	PCR test	56.6 (45.2, 68)	43	43.4 (32, 54.8)	33		61.5 (52.2, 70.8)	67	38.5 (29.2, 47.8)	42		70.6 (62.0, 79.3)	77	29.4 (20.7, 38)	32	

a. Due to a technical error, one person was not asked about their symptom attribution or test type, therefore n for those analyses is 497.

For peer review only - http://bmjopen.bmj.com/site/about/guidelines.xhtml

For peer review only

DISCUSSION

While pre-existing advice [4] and more recent messages [3] ask people to stay at home if they feel unwell, our data show that a substantial proportion of people with cough, fever or loss of their taste or smell go to work, go shopping, socialise with others, and provide care to vulnerable people. While it might be expected that such behaviours would occur less frequently in people who have a fever or who believe themselves to be infectious, we did not find this to be the case. Previous reviews have noted a wide range of factors that can prevent someone from staying at home when ill, including the absence of paid sick leave, organisational culture, a sense of professional obligation, and concern about the impact of your absence on other people.[1,2] It may be that such factors are more relevant in determining behaviour when ill than knowledge as to whether you might be contagious. If so, this has implications for interventions in this area. Encouragement to stay at home, in the absence of support, change in organisational processes, increased sick pay and indeed changes in our national culture regarding presenteeism may be insufficient.

Participants who had taken a test for COVID-19 were less likely than those who had not taken a test to have engaged in each of the five behaviours that we assessed. Given that attributing your symptoms to COVID-19 did not affect behaviour, why might this be? We have previously shown that use of a test is more common in employees who work for specific sectors where daily lateral flow tests are the norm.[8] It may be that taking a test is a marker of working in a sector where tackling the spread of infectious disease is viewed as a priority. Equally, it may be that use of a test is an indicator that someone is more adherent to Government advice in general.

Our study has several limitations. First, although we used standard market research practice to obtain our sample, we cannot be sure about its representativeness. Second, we asked people if they had developed symptoms in the past ten days, and what actions they had taken after developing symptoms. If symptoms developed ten days ago and actions were taken yesterday, the participant might have been outside of any infectious window. However, official advice is that people remain infectious with a common cold for two weeks and with influenza for eight days, suggesting this was not a major limitation.[9] Third, we relied on self-report of behaviour. If anything, the rates we identified are therefore likely to be underestimates.

For peer review only

For peer review only - http://bmjopen.bmj.com/site/about/guidelines.xhtml

ACKNOWLEDGEMENTS

We are grateful to all participants for giving up their time for this study, and to the English Department of Health and Social Care for making these data available to us.

COMPETING INTERESTS

All authors had financial support from NIHR for the submitted work; RA is an employee of the UK Health Security Agency; HWWP has received additional salary support from Public Health England and NHS England; HWWP receives consultancy fees to his employer from Ipsos MORI and has a PhD student who works at and has fees paid by Astra Zeneca; no other financial relationships with any organisations that might have an interest in the submitted work in the previous three years; no other relationships or activities that could appear to have influenced the submitted work. NTF is a participant of an independent group advising NHS Digital on the release of patient data. All authors are participants of the UK's Scientific Advisory Group for Emergencies or its subgroups.

FUNDING

This work was funded by the National Institute for Health Research (NIHR) Health Services and Delivery Research programme (NIHR project reference number (11/46/21)). Surveys were commissioned and funded by Department of Health and Social Care (DHSC), with the authors providing advice on the question design and selection. LS, RA and GJR are supported by the National Institute for Health Research Health Protection Research Unit (NIHR HPRU) in Emergency Preparedness and Response, a partnership between the UK Health Security Agency, King's College London and the University of East Anglia. RA is also supported by the NIHR HPRU in Behavioural Science and Evaluation, a partnership between the UK Health Security Agency and the University of Bristol. HWWP has received funding from Public Health England and NHS England. NTF is part funded by a grant from the UK Ministry of Defence. The views expressed are those of the authors and not necessarily those of the NIHR, Public Health England, the Department of Health and Social Care or the Ministry of Defence. The Department of Health and Social Care funded data collection (no grant number).

DATA AVAILABILITY STATEMENT

No additional data are available from the authors.

ETHICS STATEMENT

This work was conducted as a service evaluation of the Department of Health and Social Care's public communications campaign and, following advice from King's College London Research Ethics Subcommittee, was exempt from ethical approval.

AUTHOR CONTRIBUTIONS

GJR, RA, NTF, HWWP, SM, and LES conceptualized the study. LES carried out formal analyses. GJR wrote the first draft of the manuscript. RA, NTF, HWWP, SM, and LES reviewed and edited the manuscript. All authors approved the final version.

For peer review only

REFERENCES

1. Webster RK, Liu R, Hall I, Amlot R, Rubin GJ. A systematic review of infectious illness presenteeism: Prevalence, reasons and risk factors. *BMC Public Health* 2019; 19:799.

2. Daniels S, Wei H, Han Y, Catt H, Denning DW, Hall I, Regan M, Verma A, Whitfield CA, van Tongeren M. Risk factors associated with respiratory infectious disease-related presenteeism: A rapid review. MedRxiv [preprint]. https://www.medrxiv.org/content/10.1101/2021.04.12.21255302v2

3. HM Government. COVID-19 Response: Autumn and Winter Plan 2021. 14 September 2021. Available from: https://www.gov.uk/government/publications/covid-19-response-autumn-and-winter-plan-2021 (accessed 1 December 2021)

4. NHS. Flu. Available at: https://www.nhs.uk/conditions/flu/pages/introduction.aspx/amp/ (accessed 1 December 2021)

5. Rubin J, Smith L, Potts H, Fear N, Amlôt R, Michie S. Testing when symptomatic, and staying at home with influenza-like illness, during autumn and winter 2021 [30.09.2021]. Available at: https://www.gov.uk/government/publications/testing-when-symptomatic-and-staying-at-home-with-influenza-like-illness-during-autumn-and-winter-2021-30-september-2021 (accessed 1 December 2021)

6. Smith LE, Potts HWW, Amlôt R, Fear NT, Michie S, Rubin GJ. Adherence to the test, trace, and isolate system in the UK: results from 37 nationally representative surveys. *BMJ* 2021;372:n608.

7. Rubin GJ, Bakhshi S, Amlôt R, Fear N, Potts HWW & Michie S. The design of a survey questionnaire to measure perceptions and behaviour during an influenza pandemic: the Flu TElephone Survey Template (FluTEST). *Health Serv Deliv Res* 2014;2(41).

8. Smith LE, Potts HWW, Amlôt R, Fear NT, Michie S, Rubin GJ. Who is engaging with lateral flow testing for COVID-19 in the UK? The COVID-19 Rapid Survey of Adherence to Interventions and Responses (CORSAIR) study. Open Science Framework [preprint]. https://osf.io/529g7/

9. NHS. How long is someone infectious after a viral infection? Available at: https://www.nhs.uk/common-health-questions/infections/how-long-is-someone-infectious-after-a-viral-infection/ (accessed 1 December 2021)

For peer review only

For peer review only - http://bmjopen.bmj.com/site/about/guidelines.xhtml

STROBE Statement—Checklist of items that should be included in reports of ***cross-sectional studies***

	Item No	Recommendation	Page No
**Title and abstract**	1	(*a*) Indicate the study's design with a commonly used term in the title or the abstract	1
		(*b*) Provide in the abstract an informative and balanced summary of what was done and what was found	2
**Introduction**			
Background/rationale	2	Explain the scientific background and rationale for the investigation being reported	4
Objectives	3	State specific objectives, including any prespecified hypotheses	4
**Methods**			
Study design	4	Present key elements of study design early in the paper	4
Setting	5	Describe the setting, locations, and relevant dates, including periods of recruitment, exposure, follow-up, and data collection	4
Participants	6	(*a*) Give the eligibility criteria, and the sources and methods of selection of participants	4
Variables	7	Clearly define all outcomes, exposures, predictors, potential confounders, and effect modifiers. Give diagnostic criteria, if applicable	4-5
Data sources/ measurement	8*	For each variable of interest, give sources of data and details of methods of assessment (measurement). Describe comparability of assessment methods if there is more than one group	*4-5*
Bias	9	Describe any efforts to address potential sources of bias	4-5
Study size	10	Explain how the study size was arrived at	4-5
Quantitative variables	11	Explain how quantitative variables were handled in the analyses. If applicable, describe which groupings were chosen and why	4-5
Statistical methods	12	(*a*) Describe all statistical methods, including those used to control for confounding	4-5
		(*b*) Describe any methods used to examine subgroups and interactions	4-5
		(*c*) Explain how missing data were addressed	4-5
		(*d*) If applicable, describe analytical methods taking account of sampling strategy	4-5
		(*e*) Describe any sensitivity analyses	N/A
**Results**			
Participants	13*	(a) Report numbers of individuals at each stage of study—eg numbers potentially eligible, examined for eligibility, confirmed eligible, included in the study, completing follow-up, and analysed	5
		(b) Give reasons for non-participation at each stage	5
		(c) Consider use of a flow diagram	Considered
Descriptive data	14*	(a) Give characteristics of study participants (eg demographic, clinical, social) and information on exposures and potential confounders	5
		(b) Indicate number of participants with missing data for each variable of interest	5, Table 1
Outcome data	15*	Report numbers of outcome events or summary measures	

For peer review only

For peer review only - http://bmjopen.bmj.com/site/about/guidelines.xhtml

Main results	16	(*a*) Give unadjusted estimates and, if applicable, confounder-adjusted estimates and their precision (eg, 95% confidence interval). Make clear which confounders were adjusted for and why they were included	5
		(*b*) Report category boundaries when continuous variables were categorized	5
		(*c*) If relevant, consider translating estimates of relative risk into absolute risk for a meaningful time period	N/A
Other analyses	17	Report other analyses done—eg analyses of subgroups and interactions, and sensitivity analyses	N/A
**Discussion**			
Key results	18	Summarise key results with reference to study objectives	7
Limitations	19	Discuss limitations of the study, taking into account sources of potential bias or imprecision. Discuss both direction and magnitude of any potential bias	7
Interpretation	20	Give a cautious overall interpretation of results considering objectives, limitations, multiplicity of analyses, results from similar studies, and other relevant evidence	7
Generalisability	21	Discuss the generalisability (external validity) of the study results	7
**Other information**			
Funding	22	Give the source of funding and the role of the funders for the present study and, if applicable, for the original study on which the present article is based	8

*Give information separately for exposed and unexposed groups.

Note: An Explanation and Elaboration article discusses each checklist item and gives methodological background and published examples of transparent reporting. The STROBE checklist is best used in conjunction with this article (freely available on the Web sites of PLoS Medicine at http://www.plosmedicine.org/, Annals of Internal Medicine at http://www.annals.org/, and Epidemiology at http://www.epidem.com/). Information on the STROBE Initiative is available at www.strobe-statement.org.

For peer review only

BMJ Open

Do people with symptoms of an infectious illness follow advice to stay at home? Evidence from a series of cross-sectional surveys about presenteeism in the UK

Journal:	*BMJ Open*
Manuscript ID	bmjopen-2021-060511.R1
Article Type:	Original research
Date Submitted by the Author:	08-Apr-2022
Complete List of Authors:	Rubin, G James; King's College London<br>Smith, Louise; King's College London, Psychological Medicine<br>Amlot, Richard; UK Health Security Agency<br>Fear, Nicola; King's College London, ADMMH<br>Potts, Henry; University College London, Institute of Health Informatics<br>Michie, Susan; University College London, Centre for Behaviour Change
**Primary Subject Heading**:	Public health
Secondary Subject Heading:	Infectious diseases
Keywords:	COVID-19, Infection control < INFECTIOUS DISEASES, Public health < INFECTIOUS DISEASES

SCHOLARONE™
Manuscripts

I, the Submitting Author has the right to grant and does grant on behalf of all authors of the Work (as defined in the below author licence), an exclusive licence and/or a non-exclusive licence for contributions from authors who are: i) UK Crown employees; ii) where BMJ has agreed a CC-BY licence shall apply, and/or iii) in accordance with the terms applicable for US Federal Government officers or employees acting as part of their official duties; on a worldwide, perpetual, irrevocable, royalty-free basis to BMJ Publishing Group Ltd ("BMJ") its licensees and where the relevant Journal is co-owned by BMJ to the co-owners of the Journal, to publish the Work in this journal and any other BMJ products and to exploit all rights, as set out in our licence.

The Submitting Author accepts and understands that any supply made under these terms is made by BMJ to the Submitting Author unless you are acting as an employee on behalf of your employer or a postgraduate student of an affiliated institution which is paying any applicable article publishing charge ("APC") for Open Access articles. Where the Submitting Author wishes to make the Work available on an Open Access basis (and intends to pay the relevant APC), the terms of reuse of such Open Access shall be governed by a Creative Commons licence – details of these licences and which Creative Commons licence will apply to this Work are set out in our licence referred to above.

Other than as permitted in any relevant BMJ Author's Self Archiving Policies, I confirm this Work has not been accepted for publication elsewhere, is not being considered for publication elsewhere and does not duplicate material already published. I confirm all authors consent to publication of this Work and authorise the granting of this licence.

For peer review only

Do people with symptoms of an infectious illness follow advice to stay at home? Evidence from a series of cross-sectional surveys about presenteeism in the UK.

Rubin GJ[1] (0000-0002-4440-0570)*, Smith LE[1](0000-0002-1277-2564)*, Amlôt R[2] (0000-0003-3481-6588), Fear NT[1] (0000-0002-5792-2925), Potts HWW[3] (0000-0002-6200-8804), Michie S[4] (0000-0003-0063-6378).

1. Department of Psychological Medicine, Institute of Psychiatry, Psychology and Neuroscience, Weston Education Centre, King's College London, London SE5 9RJ UK

2. United Kingdom Health Security Agency, Behavioural Science Team, Porton Down, Salisbury UK

3. Institute of Health Informatics, University College London, London UK

4. Centre for Behaviour Change, University College London, London UK

Correspondence to: Professor James Rubin, Department of Psychological Medicine, Weston Education Centre, Cutcombe Road, London SE5 9RJ. Email: Gideon.rubin@kcl.ac.uk.

*Rubin and Smith contributed equally to this paper and are joint first authors

Word count: 2821

For peer review only

Abstract

Objectives: To assess the percentage of people in the UK with cough, fever or loss of taste or smell who have not had a positive COVID-19 test result who had been to work, to shops, socialised or provided care to a vulnerable person in the 10 days after developing symptoms. To investigate whether these rates differed according to the type of symptom, what the participant thought the cause of their symptoms was, and whether they had taken a COVID-19 test.

Design: Four online cross-sectional surveys using non-probability quota sampling method (n=8,547).

Setting: Data were collected across the UK from 20 September to 3 November 2021, via a market research company.

Participants: Aged over 16 years living in the UK.

Primary outcome measures: Out-of-home activity.

Results: 498 participants reported one or more symptoms and had not had a positive COVID-19 test result. Within that group, about half of employed participants had attended work while symptomatic (51.2% to 56.3% depending on the symptom, 95% CIs 42.2% to 65.6%). Rates of other contact behaviours ranged from 31.4% (caring for a vulnerable person after developing a cough: 95% CI 24.3% to 38.4%) to 61.5% (shopping for groceries or pharmacy after developing a cough: 95% CI 54.1% to 68.9%). There were no differences according to type of symptom experienced or what the participant felt might be the cause. People who had taken a COVID-19 test were less likely to go out shopping for non-essentials than people who had not taken a test.

Conclusion: Many people in the UK with symptoms of an infectious disease were not following Government advice to stay at home if they believed they had an infectious illness. Reducing these rates may require a shift in our national attitude to the acceptability of people attending work with infectious illnesses.

Keywords: behaviour, symptoms, influenza-like-illness, COVID-19

For peer review only

For peer review only - http://bmjopen.bmj.com/site/about/guidelines.xhtml

Article summary

Strengths and limitations of this study

- The use of a large (n=8,547) sample allowed identification of a large number of people who had recently experienced symptoms (n=548).
- By asking about symptoms and activities that had occurred in the past ten days, we limited the likelihood of recall bias.
- The sample was derived using a non-probability quota sampling method, hence the representativeness of the sample is unclear.
- The data are self-reported and therefore, given that it may be socially undesirable to admit attending work while ill, our reported rate of people attending work while symptomatic may be an underestimate.

For peer review only

For peer review only - http://bmjopen.bmj.com/site/about/guidelines.xhtml

INTRODUCTION

The spread of infectious disease within a workplace represents a risk to employees, those who come into contact with them and to the productivity of the organisation. Although transmission can be reduced if employees who are ill stay at home, systematic reviews have demonstrated that there are a wide range of factors that determine whether someone will have the capability, opportunity or motivation to do so.[1,2]

The COVID-19 pandemic has made this issue particularly pressing. In the UK, at the time these data were collected, people who developed a new continuous cough, a fever, or a loss or change to their sense of taste or smell (the then so-called 'cardinal symptoms' of COVID-19) were urged to stay at home and to take a polymerase chain reaction (PCR) test. If they received a positive test result, they were then legally obliged to remain at home. Yet self-isolating if you receive a positive COVID-19 result was only one aspect of the UK Government's plan to ease pressure on the National Health Service during the pandemic. According to Government guidance at the time, people should also have "[tried] to stay at home if you are feeling unwell" even in the absence of a positive COVID-19 test, while businesses were "encouraged to ask employees to stay at home if they are feeling unwell."[3] This was intended to reduce the spread of influenza-like illness throughout workplaces and reinforced pre-existing advice to stay off work or school when ill.[4]

While a growing body of research has investigated the extent to which people adhere to COVID-19 testing and self-isolation policies [5], we are unaware of evidence about the extent to which people adhere to advice to "stay at home if you are feeling unwell" even in the absence of a positive COVID-19 diagnosis. In this study, we investigated the behaviour of people who reported having symptoms, but who did not have a positive COVID-19 test result, using a series of national surveys. We analysed rates of five behaviours among people who were symptomatic: going to work, going to the shops for food or medicine, going to the shops for other things, meeting up with friends or family you do not live with, and providing help or care for a vulnerable person. We tested whether rates differed according to the symptom that was reported, what the participant believed might have caused their symptoms, and whether the participant had taken a test for COVID-19. We also assessed associations between behaviours and socio-demographic variables.

METHODS

Design

The English Department of Health and Social Care commissioned a series of cross-sectional, nationally representative surveys from market research companies, beginning in January 2020. For this analysis, we used data from survey waves 58 to 61 (20 September to 3 November 2021). More details are available elsewhere.[6, 7]

Participants

Participants were aged over 16 years, lived in the UK and had previously opted-in to receive invitations to take part in online market research surveys. Participants who took part in one wave of the Department of Health and Social Care series of surveys were prevented from taking part in any of the next three waves. We therefore elected to use the most recent four waves to ensure that each participant only contributed one set of response. The market research company sent invitations to existing panel members who met these criteria, asking them to complete the survey. Quotas were used based on age and gender (combined) and region to ensure the sample was

For peer review only - http://bmjopen.bmj.com/site/about/guidelines.xhtml

broadly representative of the UK population on these variables. Quotas were based on mid-year (2018) projections from the Office for National Statistics.

Questionnaire items

Participants were asked whether they had developed any from a list of 13 symptoms in the past ten days. This included new continuous cough, high temperature / fever, loss of sense of smell and loss of taste. Participants who reported one of these symptoms were asked "what do you think your symptoms could have been caused by" and were able to tick one or more of: "hayfever / allergies," "a cold," "asthma," "flu," "coronavirus," "long COVID," "other, please specify" [with a free-text space provided] and "don't know." They were also asked which, if any, of a list of actions they took "while you had these symptoms," which included "I took a test to confirm whether I have coronavirus." Anyone who ticked this option was asked whether they took a PCR test or a lateral flow test (LFT). Participants reporting cardinal symptoms were also asked "how many times after developing symptoms recently, if at all, have you completed each of these activities" and were presented with a list of eleven daily activities, of which five might involve contact with other people.

We asked participants for their age, gender, whether there were dependent children in their household, employment status (for those in full-, part-, or self-employment, we also asked whether they could work from home), socio-economic grade, ethnicity, first language, how many people lived in their household, vaccination status, and whether they thought or had confirmed SARS-CoV-2 infection previously. Participants were also asked three questions about their financial hardship in the last week (to what extent they had been struggling to make ends meet, skipping meals they would usually have, and were finding their current living situation difficult; Cronbach's α=.76); these items were summed to make a continuous scale, with a higher score indicated greater hardship. Participants' region was derived from their postcode.

Patient and public involvement

Lay members served on the advisory group for the project that developed our prototype survey material; this included three rounds of qualitative testing.[8] Due to the rapid nature of this research, the public was not involved in the further development of the materials during the COVID-19 pandemic.

Analysis

We restricted our sample to participants who reported having experienced one or more cardinal symptoms. We excluded anyone who reported that their most recent test for COVID-19 was positive, on the basis that such people would be legally obliged to self-isolate and should have received support and regular encouragement to do so via the UK's NHS Test and Trace service. Our sample therefore consisted of people who had received a negative test result and people who had not taken a test.

We calculated the percentage of participants who reported engaging in each of the five activities (going out to the shops for groceries/pharmacy, to shops for things other than groceries/pharmacy, to work, to meet friends or family from another household, and to provide help or care for a vulnerable person). We restricted analyses of work attendance to those who reported being in full, part-time or self-employment.

Logistic regressions (engaged in this activity, vs did not engage in this activity) were used to assess associations between activities and socio-demographic variables (survey wave, region, gender, age [raw and quadratic], ethnicity, socio-economic grade, index of multiple deprivation, presence of

For peer review only - http://bmjopen.bmj.com/site/about/guidelines.xhtml

dependent children in the household, employment status, education, English as first language, perceived immunity [composite score: been vaccinated or think had COVID-19, vs not been vaccinated and think have not had COVID-19], living alone, financial hardship).

We used logistic regressions to assess whether behaviour was associated with symptoms, attribution of symptoms (by grouping into mutually exclusive groups according to whether they reported COVID-19 as a possible cause, whether they reported another infectious disease as a possible cause [excluding COVID-19], or whether they did not list any infectious disease as a cause), and whether participants had sought a test for COVID-19 because of their recent symptoms separately. A second set of logistic regression analyses were conducted, adjusting for socio-demographic variables.

Due to the large number of analyses conducted on a single outcome (n=23), we implemented a Bonferroni correction ($p \leq 0.002$) for reporting results as being statistically significant, but we give raw p-values in the paper for completeness.

RESULTS

Out of a total sample of 8547, 548 participants (6.4%) reported cardinal symptoms. Of those, 50 reported that their most recent COVID-19 test was positive and were excluded. Of the remaining 498: 54.8% (n=273) were male, 44.8% (n=223) were female and 0.4% (n=2) preferred to self-describe or not say; 71.3% (n=355) identified as white British, 7.4% (n=37) identified as white other, 20.1% (n=100) identified as being from another ethnic group and 1.2% (n=6) preferred not to say; and 71.5% (n=365) were working, 26.7% (n=133) were not working and 1.8% (n=9) preferred not to say. The mean age was 36.3 years (standard deviation 15.2 years).

About half of employed people with fever (52.5%, 95% CI 43.5% to 61.4%), cough (51.2%, 95% CI 42.2% to 60.3%) and loss of taste or smell (56.3%, 95% CI 46.9% to 65.6%) reported having been to work after developing their symptoms. Rates of going to the shops, meeting friends or family that you do not live with, and providing care for a vulnerable person ranged from 31.4% (95% CI 24.3% to 38.4%) to 61.5% (95% CI 54.1% to 68.9%; Table 1).

Providing help or care for a vulnerable person was statistically significantly associated with having a dependent child, lesser financial hardship, and survey wave, with fewer people leaving home for this reason in later waves. See supplementary materials for full results.

Rates of out-of-home activity did not differ according to what symptom was reported or what the participant believed had caused their symptoms (Table 2). Participants who had taken a test to check whether they had COVID-19 were generally less likely to engage in each of the behaviours aside from going to work in unadjusted analyses. In adjusted analyses, only the association between going out for items other than groceries/pharmacy and having taken a test remained significant when applying our Bonferroni correction.

For peer review only - http://bmjopen.bmj.com/site/about/guidelines.xhtml

Table 1. Behaviours among people with fever, cough or loss of taste or smell and who have not had a positive COVID-19 test

		Been out to… the shops for groceries/pharmacy				the shops for things other than groceries/pharmacy				go to work [total n=356]				meet friends or family that you don't live with				provide help or care for a vulnerable person			
		No		Yes		No		Yes		No		Yes		No		Yes		No		Yes	
		% (95%CI)	*n*	% (95%CI)	*n*	% (95%CI)	*n*	% (95%CI)	*n*	% (95%CI)	*n*	% (95%CI)	*n*	% (95%CI)	*n*	% (95%CI)	*n*	% (95%CI)	*n*	% (95%CI)	*n*
Symptom	Fever	40.7 (33.2, 48.2)	68	59.3 (51.8, 66.8)	99	48.5 (40.8, 56.2)	81	51.5 (43.8, 59.2)	86	47.5 (38.6, 56.5)	58	52.5 (43.5, 61.4)	64	50.9 (43.2, 58.6)	85	49.1 (41.4, 56.8)	82	59.9 (52.4, 67.4)	100	40.1 (32.6, 47.6)	67
	Cough	38.5 (31.1, 45.9)	65	61.5 (54.1, 68.9)	104	55.6 (48.1, 63.2)	94	44.4 (36.8, 51.9)	75	48.8 (39.7, 57.8)	59	51.2 (42.2, 60.3)	62	52.7 (45.1, 60.3)	89	47.3 (39.7, 54.9)	80	68.6 (61.6, 75.7)	116	31.4 (24.3, 38.4)	53
	Loss of taste/smell	39.1 (31.5, 46.8)	63	60.9 (53.2, 68.5)	98	44.1 (36.3, 51.9)	71	55.9 (48.1, 63.7)	90	43.8 (34.4, 53.1)	49	56.3 (46.9, 65.6)	63	48.4 (40.6, 56.2)	78	51.6 (43.8, 59.4)	83	60.2 (52.6, 67.9)	97	39.8 (32.1, 47.4)	64
Attributed to[a]	COVID-19	41.6 (31.8, 51.4)	42	58.4 (48.6, 68.2)	59	48.5 (38.6, 58.4)	49	51.5 (41.6, 61.4)	52	51.2 (40.2, 62.3)	42	48.8 (37.7, 59.8)	40	53.5 (43.6, 63.4)	54	46.5 (36.6, 56.4)	47	59.4 (49.7, 69.1)	60	40.6 (30.9, 50.3)	41
	Infectious illness	40.5 (35.0, 46.0)	125	59.5 (54.0, 65.0)	184	50.8 (45.2, 56.4)	157	49.2 (43.6, 54.8)	152	43.9 (37.1, 50.6)	93	56.1 (49.4, 62.9)	119	52.8 (47.2, 58.3)	163	47.2 (41.7, 52.8)	146	64.1 (58.7, 69.5)	198	35.9 (30.5, 41.3)	111
	Non- infectious illness	33.3 (23.2, 43.4)	29	66.7 (56.6, 76.8)	58	46.0 (35.3, 56.7)	40	54.0 (43.3, 64.7)	47	50.8 (37.9, 63.7)	31	49.2 (36.3, 62.1)	30	40.2 (29.7, 50.7)	35	59.8 (49.3, 70.3)	52	63.2 (52.9, 73.6)	55	36.8 (26.4, 47.1)	32
Testing uptake[a]	No test	33.8 (28.4, 39.2)	100	66.2 (60.8, 71.6)	196	40.9 (35.2, 46.5)	121	59.1 (53.5, 64.8)	175	41.2 (34.6, 47.7)	91	58.8 (52.3, 65.4)	130	44.3 (38.6, 49.9)	131	55.7 (50.1, 61.4)	165	54.4 (48.7, 60.1)	161	45.6 (39.9, 51.3)	135
	LFT only	40.2 (30, 50.4)	37	59.8 (49.6, 70)	55	60.9 (50.7, 71)	56	39.1 (29, 49.3)	36	55.2 (42.0, 68.4)	32	44.8 (31.6, 58)	26	58.7 (48.4, 68.9)	54	41.3 (31.1, 51.6)	38	81.5 (73.4, 89.6)	75	18.5 (10.4, 26.6)	17
	PCR test	54.1 (44.6, 63.6)	59	45.9 (36.4, 55.4)	50	63.3 (54.1, 72.5)	69	36.7 (27.5, 45.9)	40	56.6 (45.2, 68)	43	43.4 (32, 54.8)	33	61.5 (52.2, 70.8)	67	38.5 (29.2, 47.8)	42	70.6 (62.0, 79.3)	77	29.4 (20.7, 38)	32

a. Due to a technical error, one person was not asked about their symptom attribution or test type, therefore n for those analyses is 497.

For peer review only - http://bmjopen.bmj.com/site/about/guidelines.xhtml

Table 2. Associations between activity and symptom, symptom attribution, and requesting a test.

		Been out to… the shops for groceries/pharmacy				the shops for things other than groceries/pharmacy				go to work				meet friends or family that you don't live with				provide help or care for a vulnerable person			
		OR for going out (95% CI) [a]	*p*	aOR for going out (95% CI) [b] †	*p*	OR for going out (95% CI) [a]	*p*	aOR for going out (95% CI) [b] †	*p*	OR for going out (95% CI) [c]	*p*	aOR for going out (95% CI) [d] †	*p*	OR for going out (95% CI) [a]	*p*	aOR for going out (95% CI) [b] †	*p*	OR for going out (95% CI) [a]	*p*	aOR for going out (95% CI) [b] †	*p*
Symptom	Fever	Ref	-	Ref	-	Ref	-	Ref	-	Ref	-	Ref	-	Ref	-	Ref	-	Ref	-	Ref	-
	Cough	1.10 (0.71 to 1.70)	0.67	1.30 (0.80 to 2.13)	0.29	0.75 (0.49 to 1.15)	0.19	0.93 (0.57 to 1.54)	0.79	0.95 (0.58 to 1.58)	0.85	1.35 (0.73 to 2.51)	0.34	0.93 (0.61 to 1.43)	0.75	0.95 (0.58 to 1.56)	0.85	0.68 (0.44 to 1.07)	0.09	0.87 (0.50 to 1.53)	0.64
	Loss of taste/smell	1.07 (0.69 to 1.66)	0.77	1.03 (0.63 to 1.7)	0.91	1.19 (0.77 to 1.84)	0.42	1.12 (0.67 to 1.86)	0.66	1.17 (0.70 to 1.95)	0.56	1.10 (0.59 to 2.04)	0.77	1.10 (0.72 to 1.70)	0.66	0.99 (0.60 to 1.62)	0.96	0.98 (0.63 to 1.53)	0.95	0.80 (0.47 to 1.37)	0.42
	Overall	$\chi^2(2)=0.2$	0.91	$\chi^2(2)=1.3$	0.52	$\chi^2(2)=4.5$	0.11	$\chi^2(2)=0.5$	0.78	$\chi^2(2)=0.6$	0.73	$\chi^2(2)=1.0$	0.62	$\chi^2(2)=0.6$	0.74	$\chi^2(2)=0.0$	0.98	$\chi^2(2)=3.5$	0.17	$\chi^2(2)=0.7$	0.72
Attributed to[a]	COVID-19	Ref	-	Ref	-	Ref	-	Ref	-	Ref	-	Ref	-	Ref	-	Ref	-	Ref	-	Ref	-
	Infectious illness	1.05 (0.66 to 1.65)	0.84	1.34 (0.80 to 2.26)	0.27	0.91 (0.58 to 1.43)	0.69	1.51 (0.90 to 2.55)	0.12	1.34 (0.81 to 2.24)	0.26	2.37 (1.26 to 4.45)	0.008	1.03 (0.66 to 1.61)	0.90	1.37 (0.82 to 2.31)	0.23	0.82 (0.52 to 1.30)	0.40	1.32 (0.76 to 2.30)	0.32
	Non-infectious illness	1.42 (0.78 to 2.58)	0.25	2.06 (1.05 to 4.05)	0.04	1.11 (0.62 to 1.97)	0.73	1.58 (0.81 to 3.07)	0.18	1.02 (0.52 to 1.97)	0.96	1.33 (0.59 to 3.02)	0.49	1.71 (0.96 to 3.05)	0.07	2.47 (1.27 to 4.80)	0.008	0.85 (0.47 to 1.54)	0.59	1.30 (0.64 to 2.63)	0.47
	Overall	$\chi^2(2)=1.7$	0.43	$\chi^2(2)=4.4$	0.11	$\chi^2(2)=0.7$	0.71	$\chi^2(2)=2.7$	0.26	$\chi^2(2)=1.8$	0.41	$\chi^2(2)=8.0$	0.02	$\chi^2(2)=4.6$	0.10	$\chi^2(2)=7.3$	0.03	$\chi^2(2)=0.7$	0.70	$\chi^2(2)=1.0$	0.60
Testing uptake[a]	No test	Ref	-	Ref	-	Ref	-	Ref	-	Ref	-	Ref	-	Ref	-	Ref	-	Ref	-	Ref	-
	LFT only	0.76 (0.47 to 1.23)	0.26	0.84 (0.48 to 1.46)	0.53	0.44 (0.28 to 0.72)	0.001*	0.58 (0.33 to 1.02)	0.06	0.57 (0.32 to 1.02)	0.06	0.87 (0.42 to 1.78)	0.70	0.56 (0.35 to 0.90)	0.02	0.70 (0.40 to 1.22)	0.20	0.27 (0.15 to 0.48)	<0.001*	0.40 (0.20 to 0.80)	0.01
	PCR test	0.43 (0.28 to 0.68)	<0.001*	0.44 (0.27 to 0.74)	0.002*	0.4 (0.25 to 0.63)	<0.001*	0.38 (0.22 to 0.65)	<0.001*	0.54 (0.32 to 0.91)	0.02	0.49 (0.25 to 0.96)	0.04	0.50 (0.32 to 0.78)	0.002*	0.47 (0.28 to 0.80)	0.005	0.50 (0.31 to 0.79)	0.004	0.43 (0.24 to 0.78)	0.005
	Overall	$\chi^2(2)=13.5$	0.001*	$\chi^2(2)=9.6$	0.008	$\chi^2(2)=21.5$	<0.001*	$\chi^2(2)=13.2$	0.001*	$\chi^2(2)=7.3$	0.03	$\chi^2(2)=4.4$	0.11	$\chi^2(2)=12.2$	0.002*	$\chi^2(2)=8.0$	0.02	$\chi^2(2)=24.3$	<0.001*	$\chi^2(2)=11.8$	0.003

A) n=497 (99.8% valid responses). B) n=462 (92.8% valid responses). C) n=355 (99.7% valid responses). D) n=337 (94.7% valid responses).

† Adjusting for all socio-demographic characteristics.

* $p \leq .002$

For peer review only

For peer review only - http://bmjopen.bmj.com/site/about/guidelines.xhtml

DISCUSSION

In the UK, while advice prior to the pandemic up [4] and subsequent messages [3] asked people to stay at home if they felt unwell, our data show that about half of people with cough, fever or loss of their sense of taste or smell, and who did not have a positive COVID-19 test result, went to work, went shopping, socialised with others, and provided care to vulnerable people.

Similar findings have been reported elsewhere. For example, one cross-sectional survey of Japanese workers identified 82 people who had experienced fever or cold symptoms between February and May 2020 [9]. Of those, 51 (62.2%) reported having been to work within seven days of symptom onset. Similarly, between 2006 and 2011, a community cohort study in England found that only a third of people with influenza-like illness reported having taken any time off work while ill [10]. This high level of 'presenteeism' is problematic. Not only is limiting the spread of infection within a workforce or community challenging if people continue to mix while symptomatic, but employees are also less likely to function effectively at work while ill [11]. Identifying ways to reduce presenteeism may pay dividends in terms of both health and economic outcomes [12].

While it might be expected that such behaviours would occur less frequently in people who have a fever or who believe themselves to be infectious, we did not find this to be the case. Previous reviews have noted a wide range of factors that can prevent someone from staying at home when ill, including the absence of paid sick leave, organisational culture, a sense of professional obligation, and concern about the impact of your absence on other people.[1,2] It may be that such factors are more relevant than knowledge as to whether you might be infectious in determining behaviour when ill. This has implications for interventions to reduce presenteeism. Encouragement to stay at home may be insufficient in the absence of support, change in organisational processes, increased sick pay and changes in our national attitudes, practices and policies regarding presenteeism.

Participants who had taken a test for COVID-19 were less likely than those who had not taken a test to have engaged in the five behaviours that we assessed in unadjusted analyses, although only the association between going out for items other than groceries/pharmacy and having taken a test remained significant after correcting for multiple comparisons. Given that attributing your symptoms to COVID-19 did not affect behaviour, why might this be? We have previously shown that use of a test is more common in employees who work for specific sectors where daily lateral flow tests are the norm.[13] It may be that taking a test is a marker of working in a sector where tackling the spread of infectious disease is viewed as a priority. It may also be that using tests indicates adherence to Government advice in general.

We found relatively few associations between sociodemographic variables and contact behaviours when symptomatic, suggesting that these behaviours are determined by factors in common across the UK. Three exceptions stand out. First, participants with dependent children were more likely to leave home when ill in order to care for a vulnerable person. We speculate that having dependent children at home may also be associated with having elderly relatives (e.g. grandparents) who may require care. Second, participants who reported greater financial hardship were less likely to leave home to care for vulnerable people. We are not clear why this is. Third, participants in later survey waves were generally less likely to report leaving home than those in the first wave. At the time of our data collection, levels of contact behaviours outside of the home were relatively stable across the population [14]. It is possible that our finding is because the first wave of our data collection coincided with the start of a new academic year, with parents and students feeling more obliged to leave home that week than normal.

For peer review only - http://bmjopen.bmj.com/site/about/guidelines.xhtml

Throughout the COVID-19 pandemic, a number of studies have focused on whether people who have COVID-19 adhere to advice to self-isolate. This work has identified a range of factors that may reduce adherence, including the absence of symptoms [15], having alternative explanations for symptoms [16], financial challenges [17] and the emotional impact of isolation [18]. However, the context of these studies makes it challenging to generalise from them to the issue of staying at home for a non-COVID-19 infectious illness. For example, in the UK at the time of our data collection people who received a positive COVID-19 test result were legally obliged to self-isolate, were eligible for statutory sick pay from the first day of their illness, as well as an additional support payment if they were on a low income, received regular support calls and text messages from a national service throughout their self-isolation, and could access a range of support packages including food deliveries, dog walking and other services depending on provision in their local area. In contrast, people with influenza-like illness who did not have a positive COVID-19 test result were only eligible for statutory sick pay starting from the fourth day of their illness; no additional support was provided.

As part of the UK Government's plan to "live with" SARS-CoV-2, advice from Ministers has been for members of the public treat COVID-19 in the same way as "all other infectious diseases." [19] As testing and support mechanisms for COVID-19 are withdrawn, identifying approaches to reduce the impact of undifferentiated acute respiratory illnesses will become ever more important. To our knowledge, there have been no randomized controlled trials or natural experiments evaluating potential approaches to reduce attendance at work by people with an infectious illness. This is likely to be a fruitful avenue of research for future studies.

Strengths and limitations

The use of a large sample (n=8,547) allowed us to identify a reasonably large subset of people who had experienced recent symptoms in the absence of a positive COVID-19 test result (n= 498). By asking this group about behaviours that had occurred relatively recently, we reduced the impact of recall bias. Nonetheless, our study has several limitations. First, although we used standard market research practice to obtain our sample, we cannot be sure about its representativeness. Second, we asked people if they had developed symptoms in the past ten days, and what actions they had taken after developing symptoms. If symptoms developed ten days ago and actions were taken yesterday, the participant might have been outside their infectious window. However, official advice is that people remain infectious with a common cold for two weeks and with influenza for eight days, suggesting this was not a major limitation.[20] Third, we relied on self-report of behaviour. Given that attending work, socialising and caring for vulnerable people while symptomatic may be socially undesirable to admit, the rates we identified for these outcomes are likely to be underestimates.

Conclusion

Substantial numbers of people who have symptoms of an infectious illness attend work, go shopping, socialise with others, and provide care to vulnerable people. This occurs at a high rate even when people suspect that they may be infectious. As SARS-CoV-2 adds to the burden of infectious diseases on populations, a greater focus on developing and evaluating ways to reduce presenteeism is more important than ever.

For peer review only - http://bmjopen.bmj.com/site/about/guidelines.xhtml

ACKNOWLEDGEMENTS

We are grateful to all participants for giving up their time for this study, and to the English Department of Health and Social Care for making these data available to us.

COMPETING INTERESTS

All authors had financial support from NIHR for the submitted work; RA is an employee of the UK Health Security Agency; HWWP has received additional salary support from Public Health England and NHS England; HWWP receives consultancy fees to his employer from Ipsos MORI and has a PhD student who works at and has fees paid by Astra Zeneca; at the time of writing GJR is acting as an expert witness in an unrelated case involving Bayer PLC, supported by LS; no other financial relationships with any organisations that might have an interest in the submitted work in the previous three years; no other relationships or activities that could appear to have influenced the submitted work. NTF is a participant of an independent group advising NHS Digital on the release of patient data. At the time of writing this paper, all authors were participants of the UK's Scientific Advisory Group for Emergencies or its subgroups.

FUNDING

This work was funded by the National Institute for Health Research (NIHR) Health Services and Delivery Research programme (NIHR project reference number (11/46/21)). Surveys were commissioned and funded by Department of Health and Social Care (DHSC), with the authors providing advice on the question design and selection. LS, RA and GJR are supported by the National Institute for Health Research Health Protection Research Unit (NIHR HPRU) in Emergency Preparedness and Response, a partnership between the UK Health Security Agency, King's College London and the University of East Anglia. RA is also supported by the NIHR HPRU in Behavioural Science and Evaluation, a partnership between the UK Health Security Agency and the University of Bristol. HWWP has received funding from Public Health England and NHS England. NTF is part funded by a grant from the UK Ministry of Defence. The views expressed are those of the authors and not necessarily those of the NIHR, Public Health England, the Department of Health and Social Care or the Ministry of Defence. The Department of Health and Social Care funded data collection (no grant number).

DATA AVAILABILITY STATEMENT

No additional data are available from the authors.

ETHICS STATEMENT

This work was conducted as a service evaluation of the Department of Health and Social Care's public communications campaign and, following advice from King's College London Research Ethics Subcommittee, was exempt from ethical approval.

AUTHOR CONTRIBUTIONS

GJR, RA, NTF, HWWP, SM, and LES conceptualized the study. LES carried out formal analyses. GJR wrote the first draft of the manuscript. RA, NTF, HWWP, SM, and LES reviewed and edited the manuscript. All authors approved the final version.

For peer review only

REFERENCES

1. Webster RK, Liu R, Hall I, Amlot R, Rubin GJ. A systematic review of infectious illness presenteeism: Prevalence, reasons and risk factors. *BMC Public Health* 2019; 19:799.

2. Daniels S, Wei H, Han Y, Catt H, Denning DW, Hall I, Regan M, Verma A, Whitfield CA, van Tongeren M. Risk factors associated with respiratory infectious disease-related presenteeism: A rapid review. MedRxiv [preprint]. https://www.medrxiv.org/content/10.1101/2021.04.12.21255302v2

3. HM Government. COVID-19 Response: Autumn and Winter Plan 2021. 14 September 2021. Available from: https://www.gov.uk/government/publications/covid-19-response-autumn-and-winter-plan-2021 (accessed 1 December 2021)

4. NHS. Flu. Available at: https://www.nhs.uk/conditions/flu/pages/introduction.aspx/amp/ (accessed 1 December 2021)

5. Rubin J, Smith L, Potts H, Fear N, Amlôt R, Michie S. Testing when symptomatic, and staying at home with influenza-like illness, during autumn and winter 2021 [30.09.2021]. Available at: https://www.gov.uk/government/publications/testing-when-symptomatic-and-staying-at-home-with-influenza-like-illness-during-autumn-and-winter-2021-30-september-2021 (accessed 1 December 2021)

6. Smith LE, Potts HWW, Amlôt R, Fear NT, Michie S, Rubin GJ. Adherence to the test, trace, and isolate system in the UK: results from 37 nationally representative surveys. *BMJ* 2021;372:n608.

7. http://epr.hpru.nihr.ac.uk/our-research/research-themes/response/corsair-study [Accessed 26 March 2022]

8. Rubin GJ, Bakhshi S, Amlôt R, Fear N, Potts HWW & Michie S. The design of a survey questionnaire to measure perceptions and behaviour during an influenza pandemic: the Flu TElephone Survey Template (FluTEST). *Health Serv Deliv Res* 2014;2(41).

9. Machida M, Nakamura I, Saito R, Nakaya T, Hanibuchi T, Takamiya T, Odagiri Y, Fukushima H, Amagasa S, Kojima T, Watanabe H, Inoue S. The actual implementation status of self-isolation among Japanese workers during the COVID-19 outbreak. *Tropical Medicine and Health* 2020; 48: 63

10. Fragaszy EB, Warren-Gash C, White PJ, Zambon M, Edmunds WJ, Nguyen-Van-Tam JS, Hayward AC. Effects of seasonal and pandemic influenza on health-related quality of life, work and school absence in England. Results from the Flu Watch cohort study. *Influenza and Other Respiratory Viruses* 2018;12(1): 171-182.

11. Dicpinigatis PV, Eccles R, Blaiss MS, Wingertzahn MA. Impact of cough and common cold on productivity, absenteeism, and daily life in the United States: ACHOO survey. *Curr Med Res Opin* 2015; 31(8): 1519-1525.

12. Stearns J, White C. Can paid sick leave mandates reduce leave-taking? Labour Economics 2018;51: 227-246.

13. Smith LE, Potts HWW, Amlôt R, Fear NT, Michie S, Rubin GJ. Who is engaging with lateral flow testing for COVID-19 in the UK? The COVID-19 Rapid Survey of Adherence to Interventions and Responses (CORSAIR) study. *BMJ Open* 2022; 12: e058060.

For peer review only - http://bmjopen.bmj.com/site/about/guidelines.xhtml

14. Smith LE, Potts HWW, Amlot R, Fear NT, Michie S, Rubin GJ. Engagement with protective behaviours in the UK during the COVID-19 pandemic: A series of cross-sectional surveys (the COVID-19 rapid survey of adherence to interventions and responses [CORSAIR] study). *BMC Public Health* 2022; 22: 472.

15. Steens A, de Blasio BF, Veneti L, Gimma A, Edmunds WJ, Van Zandvoort K, Jarvis CI, Forland F, Robberstad B. Poor self-reported adherence to COVID-19-related quarantine / isolation requests, Norway, April to July 2020. *Eurosurveillance* 2020; 25(37).

16. Mowbray F, Woodland L, Smith LE, Amlot R, Rubin GJ. Is my cough a cold of covid? A qualitative study of COVID-19 symptoms recognition and attitudes toward testing in the UK. *Frontiers in Public Health* 2021; 9: 716421.

17. McAllister F, Blunt A, Davies C. Financial challenges linked to self-isolation and perceptions of the Self-Isolation Support Scheme in Wales: qualitative findings. 2022. Cardiff: Welsh Government, GSR report number 21/2022.

18. Smith LE, Amlot R, Lambert H, Oliver I, Robin C, Yardley L, Rubin GJ. Factors associated with adherence to self-isolation and lockdown measures in the UK: A cross-sectional survey. *Public Health* 2020; 187: 41-52.

19. Smyth, C. Sajid Javid says rise in COVID cases no cause for concern. The Times, 21 March 2022.

20. NHS. How long is someone infectious after a viral infection? Available at: https://www.nhs.uk/common-health-questions/infections/how-long-is-someone-infectious-after-a-viral-infection/ (accessed 1 December 2021)

For peer review only

For peer review only - http://bmjopen.bmj.com/site/about/guidelines.xhtml

SUPPLEMENTARY MATERIALS

Table 1. Socio-demographic characteristics associated with leaving home for different reasons while symptomatic.

Attribute	Level	*Been out to…* the shops for groceries/pharmacy [a] aOR for going out (95% CI) †	*p*	the shops for things other than groceries/pharmacy [b] aOR for going out (95% CI) †	*p*	go to work [c] aOR for going out (95% CI) †	*p*	meet friends or family that you don't live with [d] aOR for going out (95% CI) †	*p*	provide help or care for a vulnerable person [e] aOR for going out (95% CI) †	*p*
Survey wave in timepoint	20 to 22 September 2021 (wave 58)	Ref	-	Ref	-	Ref	-	Ref	-	Ref	-
	4 to 6 October 2021 (wave 59)	0.47 (0.26 to 0.84)	0.01	0.39 (0.22 to 0.70)	0.001*	0.44 (0.21 to 0.90)	0.02	0.48 (0.27 to 0.86)	0.01	0.43 (0.24 to 0.79)	0.007
	18 to 20 October 2021 (wave 60)	0.37 (0.21 to 0.68)	0.001*	0.41 (0.23 to 0.75)	0.003	0.32 (0.16 to 0.65)	0.002*	0.37 (0.21 to 0.67)	0.001*	0.35 (0.18 to 0.65)	0.001*
	1 to 4 November 2021 (wave 61)	0.55 (0.30 to 1.00)	0.05	0.52 (0.29 to 0.93)	0.03	0.33 (0.16 to 0.69)	0.003	0.41 (0.23 to 0.73)	0.002*	0.32 (0.17 to 0.61)	0.001*
	Overall	$\chi^2(3)$=11.3	0.01	$\chi^2(3)$=12.4	0.006	$\chi^2(3)$=12.3	0.006	$\chi^2(3)$=13.4	0.004	$\chi^2(3)$=16.0	0.001*
Region	North (North West, North East, Yorkshire and the Humber)	Ref	-	Ref	-	Ref	-	Ref	-	Ref	-
	Midlands (East Midlands, West Midlands, East of England)	0.89 (0.50 to 1.58)	0.69	0.97 (0.54 to 1.75)	0.93	0.93 (0.45 to 1.92)	0.84	0.75 (0.42 to 1.34)	0.34	0.59 (0.31 to 1.13)	0.11
	London	1.33 (0.68 to 2.61)	0.40	1.51 (0.77 to 2.95)	0.23	1.71 (0.74 to 3.97)	0.21	1.19 (0.62 to 2.29)	0.61	0.75 (0.37 to 1.53)	0.43
	South (South East, South West)	1.54 (0.81 to 2.92)	0.18	1.21 (0.64 to 2.28)	0.55	1.79 (0.82 to 3.91)	0.14	0.92 (0.49 to 1.72)	0.80	1.02 (0.51 to 2.01)	0.96
	Scotland, Wales, Northern Ireland	0.78 (0.41 to 1.49)	0.46	0.67 (0.34 to 1.30)	0.23	0.74 (0.32 to 1.70)	0.47	0.62 (0.32 to 1.20)	0.16	0.38 (0.17 to 0.83)	0.01
	Overall	$\chi^2(4)$=5.4	0.25	$\chi^2(4)$=5.2	0.26	$\chi^2(4)$=6.7	0.15	$\chi^2(4)$=4.3	0.37	$\chi^2(4)$=8.4	0.08
Gender	Male	Ref	-	Ref	-	Ref	-	Ref	-	Ref	-
	Female	0.78 (0.52 to 1.17)	0.22	0.75 (0.49 to 1.13)	0.16	0.55 (0.33 to 0.93)	0.03	1.09 (0.73 to 1.65)	0.67	0.67 (0.43 to 1.07)	0.09
Age (per decade)	Raw age	1.00 (0.98 to 1.02)	0.65	0.99 (0.97 to 1.01)	0.30	0.96 (0.94 to 0.99)	0.01	0.98 (0.96 to 1.00)	0.04	0.97 (0.95 to 0.99)	0.01
Age: quadratic (age-mean)^2	-	1.0001 (0.9996 to 1.0007)	0.65	1.0005 (0.9998 to 1.0012)	0.14	1.0011 (0.9998 to 1.0024)	0.11	1.0005 (0.9999 to 1.0011)	0.09	1.0004 (0.9998 to 1.001)	0.16
Dependent child in household	None	Ref	-	Ref	-	Ref	-	Ref	-	Ref	-
	Child present	1.20 (0.76 to 1.90)	0.43	1.78 (1.11 to 2.84)	0.02	2.50 (1.38 to 4.52)	0.003	1.64 (1.03 to 2.59)	0.04	2.85 (1.70 to 4.78)	<0.001*
Employment status	Not employed	Ref	-	Ref	-	-	-	Ref	-	Ref	-
	Employed, can work from home all the time	1.39 (0.81 to 2.39)	0.23	1.51 (0.87 to 2.63)	0.14	Ref	-	1.41 (0.83 to 2.42)	0.21	1.70 (0.93 to 3.12)	0.08
	Employed, cannot work from home all the time	1.00 (0.60 to 1.65)	0.99	1.24 (0.73 to 2.09)	0.43	1.26 (0.75 to 2.09)	0.38	0.80 (0.48 to 1.34)	0.40	1.39 (0.77 to 2.51)	0.27
	Overall	$\chi^2(2)$=2.2	0.33	$\chi^2(2)$=2.2	0.34	-	-	$\chi^2(2)$=5.5	0.06	$\chi^2(2)$=3.0	0.22
Socio-economic grade‡	ABC1	Ref	-	Ref	-	Ref	-	Ref	-	Ref	-
	C2DE	1.06 (0.71 to 1.59)	0.77	1.08 (0.72 to 1.63)	0.71	1.84 (1.10 to 3.07)	0.02	1.44 (0.96 to 2.17)	0.08	1.27 (0.81 to 1.99)	0.31
Financial hardship	Range 3 (least) to 15 (most)	0.98 (0.91 to 1.05)	0.50	0.91 (0.85 to 0.98)	0.01	0.92 (0.85 to 1.00)	0.05	0.97 (0.91 to 1.05)	0.48	0.86 (0.79 to 0.94)	<0.001*
Ethnicity	White British	Ref	-	Ref	-	Ref	-	Ref	-	Ref	-
	White other	0.99 (0.42 to 2.35)	0.98	1.30 (0.54 to 3.11)	0.55	0.81 (0.30 to 2.22)	0.69	0.76 (0.32 to 1.80)	0.53	1.57 (0.63 to 3.91)	0.33
	Black and minority ethnicity	1.03 (0.57 to 1.85)	0.92	1.04 (0.58 to 1.86)	0.90	0.65 (0.33 to 1.31)	0.23	1.04 (0.58 to 1.84)	0.90	1.48 (0.81 to 2.72)	0.21
	Overall	$\chi^2(2)$=0.0	0.99	$\chi^2(2)$=0.4	0.84	$\chi^2(2)$=1.4	0.49	$\chi^2(2)$=0.5	0.78	$\chi^2(2)$=1.9	0.38
First language	Not English	Ref	-	Ref	-	Ref	-	Ref	-	Ref	-
	English	1.20 (0.65 to 2.19)	0.56	1.37 (0.75 to 2.52)	0.31	1.15 (0.55 to 2.40)	0.70	1.13 (0.62 to 2.07)	0.69	0.95 (0.50 to 1.79)	0.87

For peer review only

Living alone	Not living alone	Ref	-	Ref	-	Ref	-	Ref	-	Ref	-
	Living alone	0.80 (0.43 to 1.49)	0.49	0.75 (0.39 to 1.44)	0.39	0.70 (0.31 to 1.59)	0.40	1.06 (0.57 to 1.99)	0.85	0.67 (0.30 to 1.49)	0.32
Vaccination status	Not vaccinated	Ref	-	Ref	-	Ref	-	Ref	-	Ref	-
	1 dose	1.29 (0.65 to 2.54)	0.47	1.06 (0.55 to 2.06)	0.86	1.87 (0.81 to 4.32)	0.14	1.35 (0.70 to 2.61)	0.37	1.37 (0.69 to 2.70)	0.37
	2 doses or more	0.90 (0.54 to 1.49)	0.68	0.77 (0.46 to 1.28)	0.31	1.17 (0.63 to 2.17)	0.63	0.75 (0.46 to 1.24)	0.26	0.78 (0.46 to 1.34)	0.37
	Overall	$\chi^2(2)=1.2$	0.54	$\chi^2(2)=1.5$	0.47	$\chi^2(2)=2.2$	0.34	$\chi^2(2)=3.7$	0.16	$\chi^2(2)=2.9$	0.23
Ever had COVID-19	Think not	Ref	-	Ref	-	Ref	-	Ref	-	Ref	-
	Think yes	1.33 (0.88 to 2.01)	0.17	1.68 (1.11 to 2.55)	0.01	1.50 (0.91 to 2.49)	0.11	1.19 (0.79 to 1.79)	0.41	1.19 (0.76 to 1.86)	0.46

A) n=462 (92.8% valid responses). B) n=462 (92.8% valid responses). C) n=337 (94.7% valid responses). D) n=462 (92.8% valid responses). E) n=462 (92.8% valid responses).

† Adjusting for all other socio-demographic characteristics.

* $p \leq .002$

For peer review only

STROBE Statement—Checklist of items that should be included in reports of ***cross-sectional studies***

	Item No	Recommendation	Page No (clean manuscript)
**Title and abstract**	1	(*a*) Indicate the study's design with a commonly used term in the title or the abstract	1
		(*b*) Provide in the abstract an informative and balanced summary of what was done and what was found	2
**Introduction**			
Background/rationale	2	Explain the scientific background and rationale for the investigation being reported	4
Objectives	3	State specific objectives, including any prespecified hypotheses	4
**Methods**			
Study design	4	Present key elements of study design early in the paper	4
Setting	5	Describe the setting, locations, and relevant dates, including periods of recruitment, exposure, follow-up, and data collection	4
Participants	6	(*a*) Give the eligibility criteria, and the sources and methods of selection of participants	4-5
Variables	7	Clearly define all outcomes, exposures, predictors, potential confounders, and effect modifiers. Give diagnostic criteria, if applicable	5
Data sources/ measurement	8*	For each variable of interest, give sources of data and details of methods of assessment (measurement). Describe comparability of assessment methods if there is more than one group	*5-6*
Bias	9	Describe any efforts to address potential sources of bias	4-6
Study size	10	Explain how the study size was arrived at	4-6
Quantitative variables	11	Explain how quantitative variables were handled in the analyses. If applicable, describe which groupings were chosen and why	5-6
Statistical methods	12	(*a*) Describe all statistical methods, including those used to control for confounding	5-6
		(*b*) Describe any methods used to examine subgroups and interactions	5-6
		(*c*) Explain how missing data were addressed	5-6
		(*d*) If applicable, describe analytical methods taking account of sampling strategy	5-6
		(*e*) Describe any sensitivity analyses	N/A
**Results**			
Participants	13*	(a) Report numbers of individuals at each stage of study—eg numbers potentially eligible, examined for eligibility, confirmed eligible, included in the study, completing follow-up, and analysed	6
		(b) Give reasons for non-participation at each stage	6
		(c) Consider use of a flow diagram	Considered
Descriptive data	14*	(a) Give characteristics of study participants (eg demographic, clinical, social) and information on exposures and potential confounders	6

For peer review only - http://bmjopen.bmj.com/site/about/guidelines.xhtml

		(b) Indicate number of participants with missing data for each variable of interest	6, Table 1
Outcome data	15*	Report numbers of outcome events or summary measures	6, Table 1
Main results	16	(*a*) Give unadjusted estimates and, if applicable, confounder-adjusted estimates and their precision (eg, 95% confidence interval). Make clear which confounders were adjusted for and why they were included	6, Tables 1 and 2
		(*b*) Report category boundaries when continuous variables were categorized	6
		(*c*) If relevant, consider translating estimates of relative risk into absolute risk for a meaningful time period	N/A
Other analyses	17	Report other analyses done—eg analyses of subgroups and interactions, and sensitivity analyses	N/A
**Discussion**			
Key results	18	Summarise key results with reference to study objectives	9
Limitations	19	Discuss limitations of the study, taking into account sources of potential bias or imprecision. Discuss both direction and magnitude of any potential bias	10
Interpretation	20	Give a cautious overall interpretation of results considering objectives, limitations, multiplicity of analyses, results from similar studies, and other relevant evidence	9-10
Generalisability	21	Discuss the generalisability (external validity) of the study results	9-10
**Other information**			
Funding	22	Give the source of funding and the role of the funders for the present study and, if applicable, for the original study on which the present article is based	11

*Give information separately for exposed and unexposed groups.

Note: An Explanation and Elaboration article discusses each checklist item and gives methodological background and published examples of transparent reporting. The STROBE checklist is best used in conjunction with this article (freely available on the Web sites of PLoS Medicine at http://www.plosmedicine.org/, Annals of Internal Medicine at http://www.annals.org/, and Epidemiology at http://www.epidem.com/). Information on the STROBE Initiative is available at www.strobe-statement.org.

For peer review only

For peer review only - http://bmjopen.bmj.com/site/about/guidelines.xhtml
